# Supplementary material for: Interaction of Conazole Pesticides Epoxiconazole and Prothioconazole with Human and Bovine Serum Albumin Studied Using Spectroscopic Methods and Molecular Modeling
Source: Int J Mol Sci. 2021 Feb 15;22(4):1925. doi: 10.3390/ijms22041925 (PMC7919476; doi:10.3390/ijms22041925)
Supplement: Supplementary file 1 [file ijms-22-01925-s001.pdf]

Supplementary Material

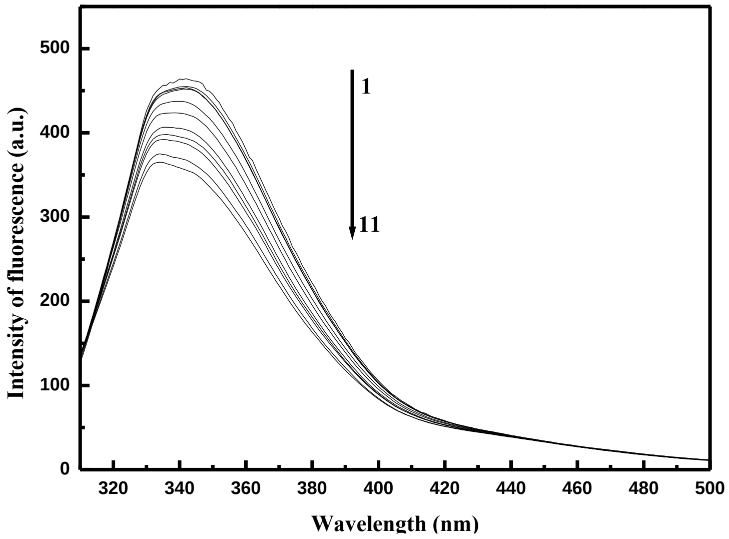

Figure S1 Quenching of fluorescence of HSA after binding EPX. c (HSA) = 2×10^{-6} mol/L; c (EPX) = 2×10^{-6} mol/L–32×10^{-6} mol/L (1–11).

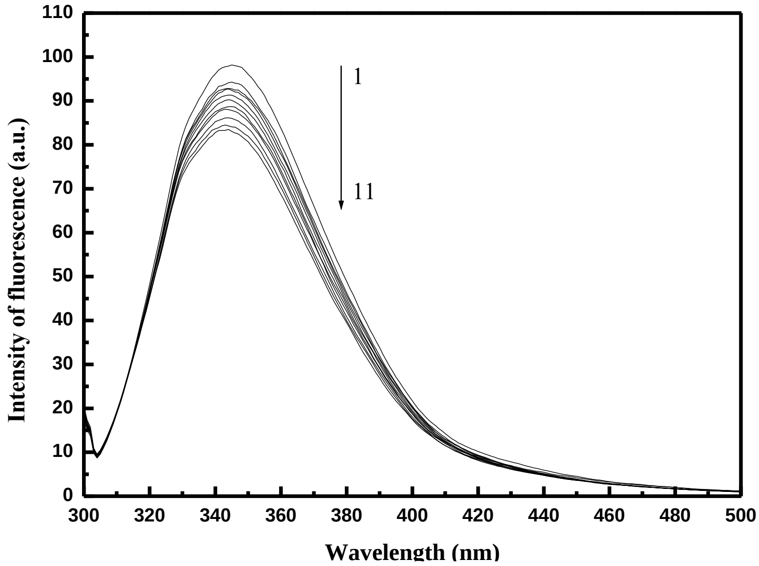

Figure S2 Quenching of fluorescence of BSA after binding EPX. c (BSA) = 2×10^{-6} mol/L; c (EPX) = 2×10^{-6} mol/L–32×10^{-6} mol/L (1–11).

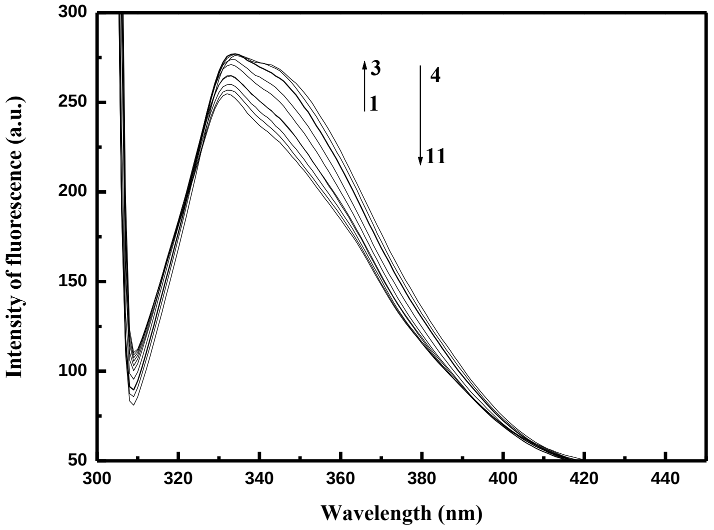

Figure S3 Quenching of fluorescence of HSA after binding PTC. c (HSA) = 2×10^{-6} mol/L; c (PTC) = 2×10^{-6} mol/L–32×10^{-6} mol/L (1–11).

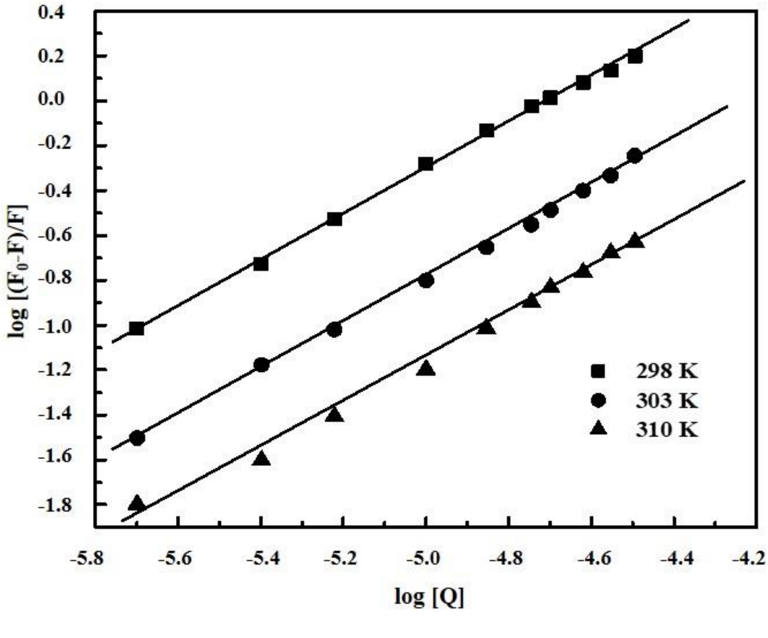

Figure S4 Hill plot for EPX/HSA interaction. T = 298 K, 303 K and 310 K.

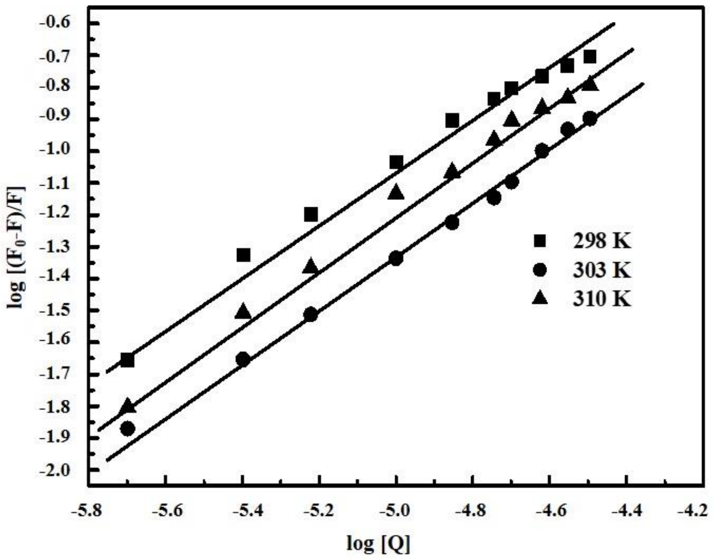

Figure S5 Hill plot for EPX/BSA interaction. T = 298 K, 303 K and 310 K.

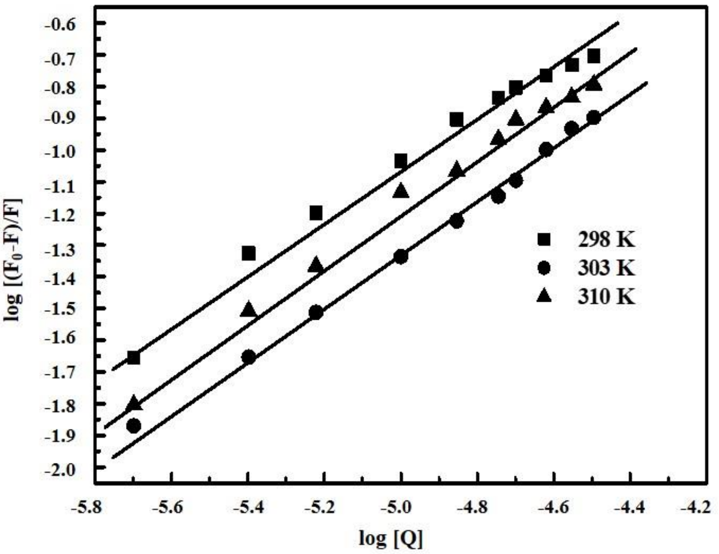

Figure S6 Hill plot for PTC/HSA interaction. T = 298 K, 303 K and 310 K.

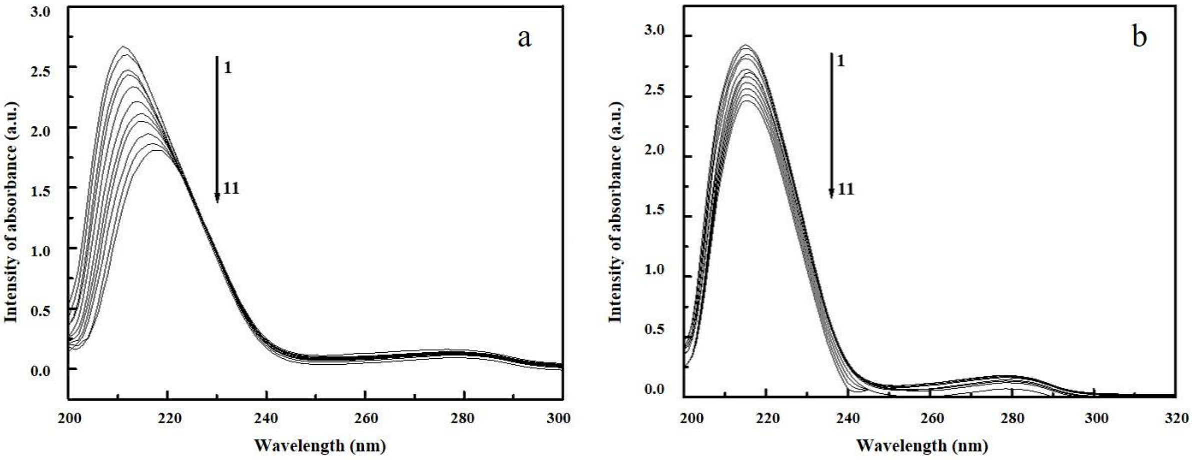

Figure 7. UV/VIS absorption spectra of BSA in the presence of EPX (**a**) and PTC (**b**). c (BSA) = 2×10^{-6} mol/L;c (EPX, PTC) = 2×10^{-6} mol/L–32×10^{-6} mol/L (1–11).

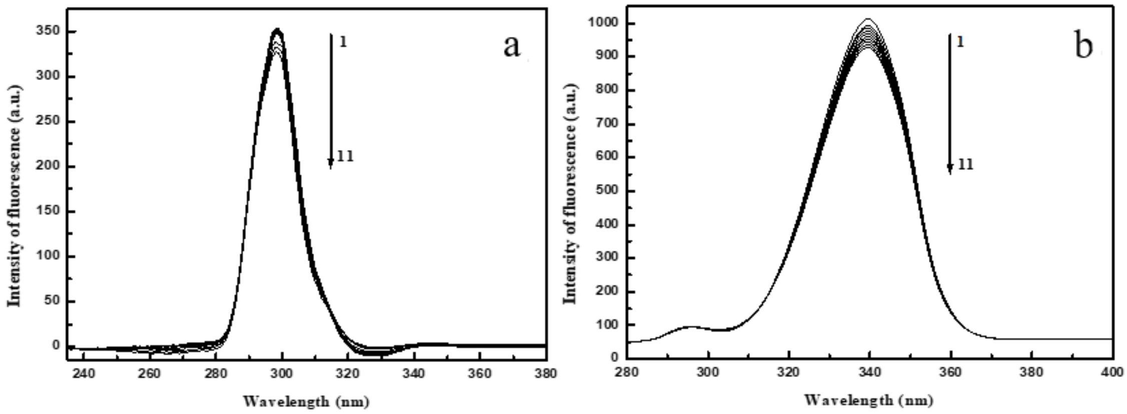

Figure S8 The synchronous spectra of Tyr (**a**) and Trp (**b**) of HSA in the presence of EPX. c (HSA) = 2×10^{-6} mol/L; c (EPX) =2×10^{-6} mol/L–32×10^{-6} mol/L (1–11)

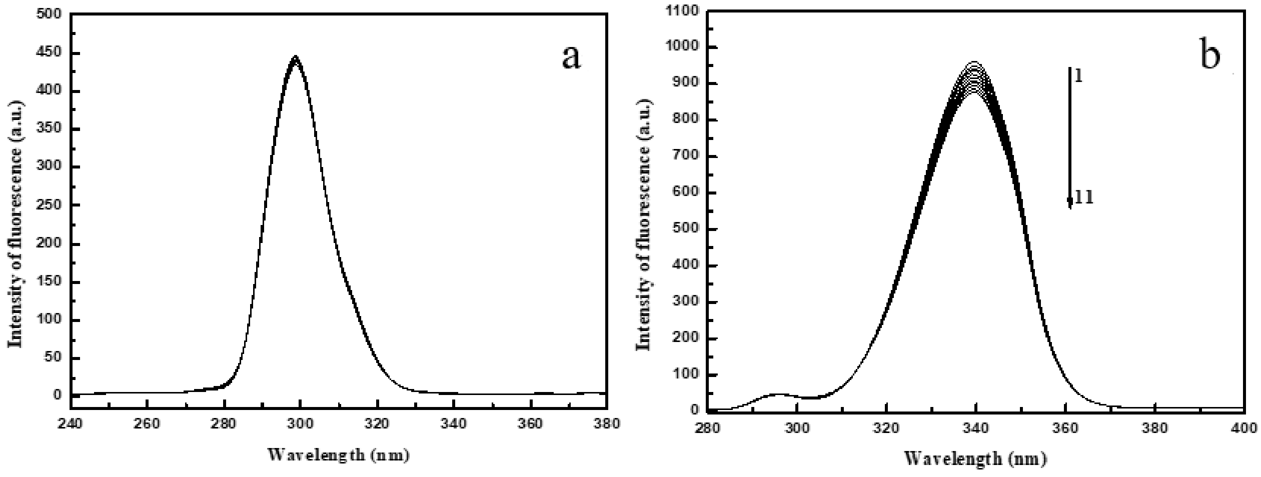

Figure S9 The synchronous spectra of Tyr (**a**) and Trp (**b**) of BSA in the presence of EPX. c (BSA) = 2×10^{-6} mol/L; c (EPX) = 2×10^{-6} mol/L–32×10^{-6} mol/L (1–11)

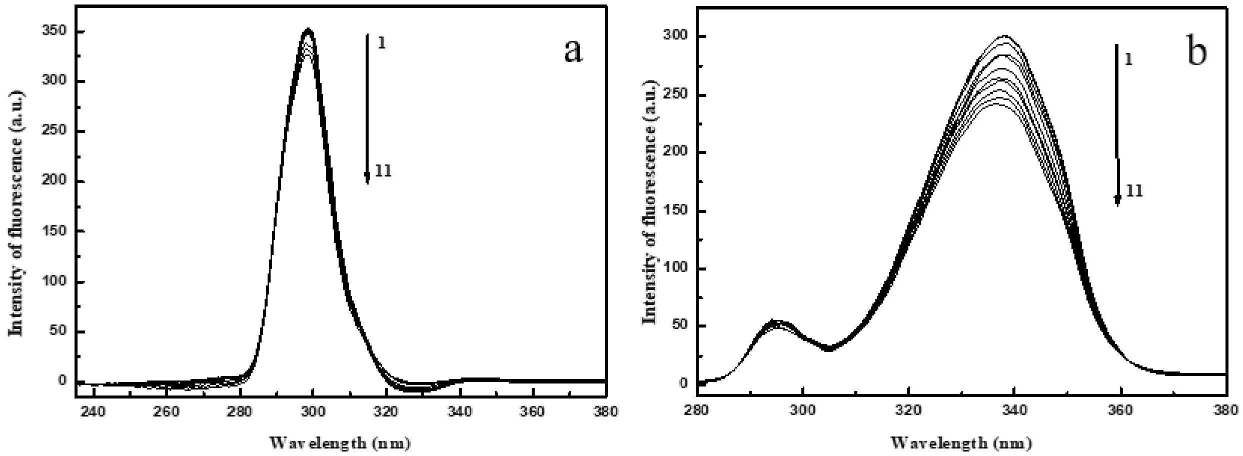

Figure S10 The synchronous spectra of Tyr (**a**) and Trp (**b**) of HSA in the presence of PTC. c (HSA) = 2×10^{-6} mol/L; c (PTC) = 2×10^{-6} mol/L–32×10^{-6} mol/L (1–11)

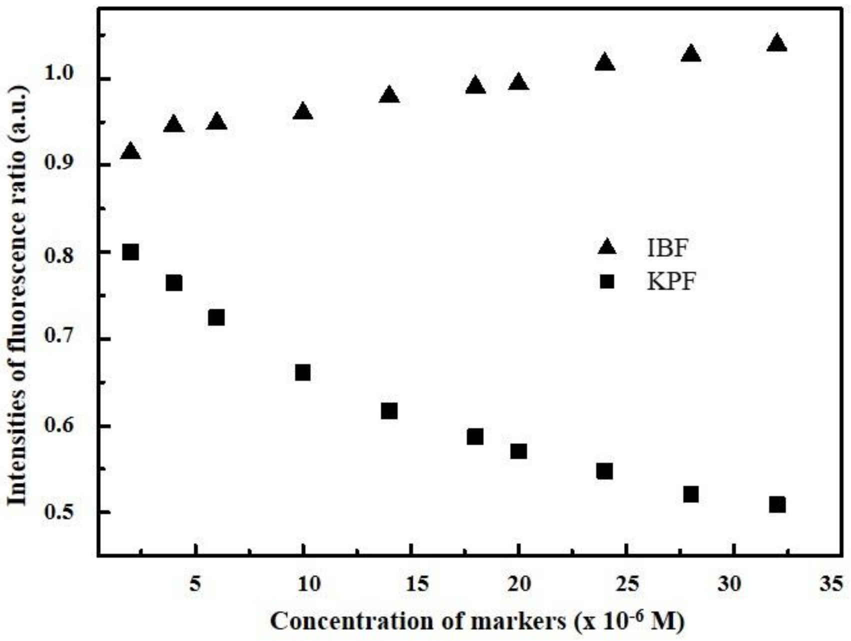

Figure S11 Intensities of fluorescence ratio of EPX/HSA complex in the presence of KPF and IBF markers

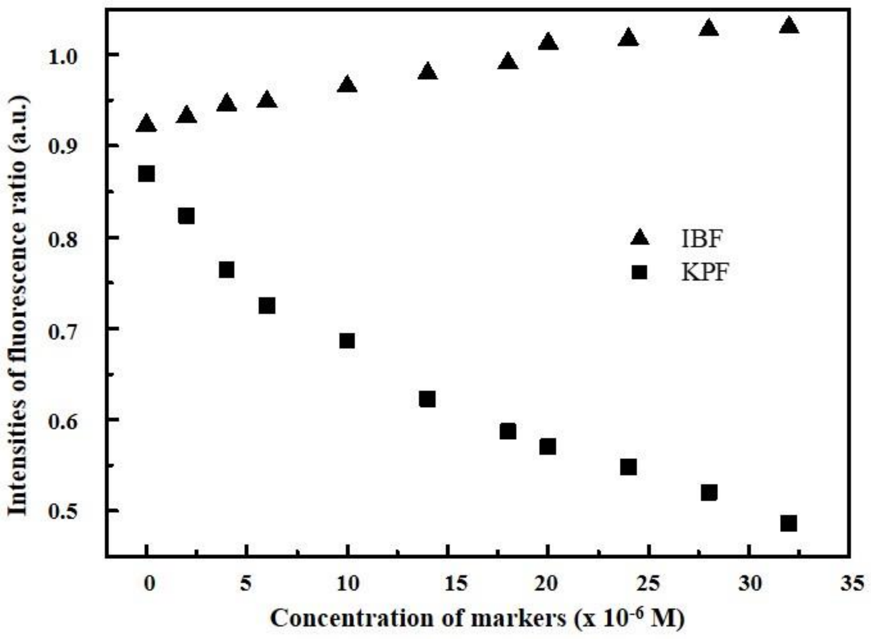

Figure S12 Intensities of fluorescence ratio of EPX/BSA complex in the presence of KPF and IBF markers

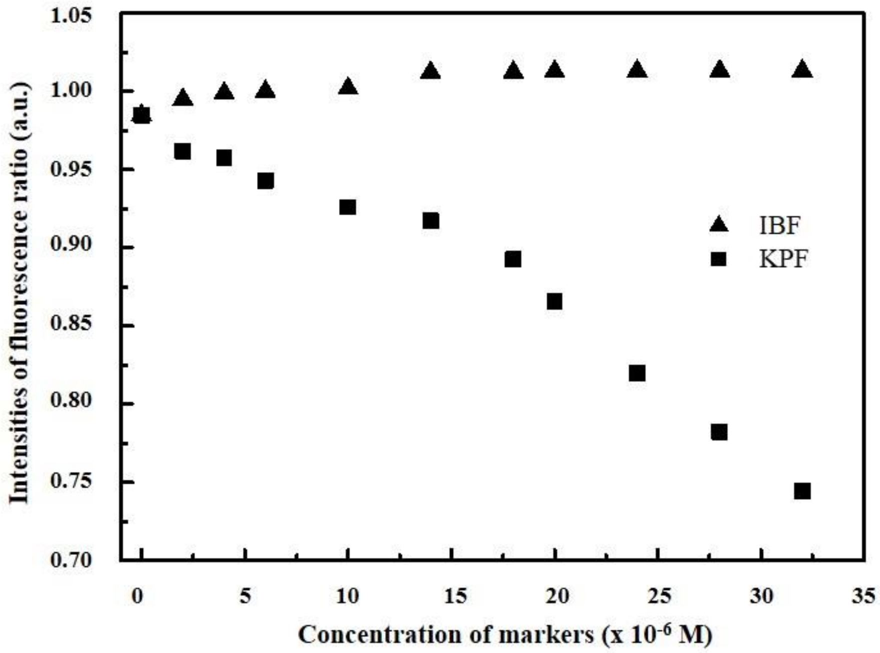

Figure S13 Intensities of fluorescence ratio of PTC/HSA complex in the presence of KPF and IBF markers

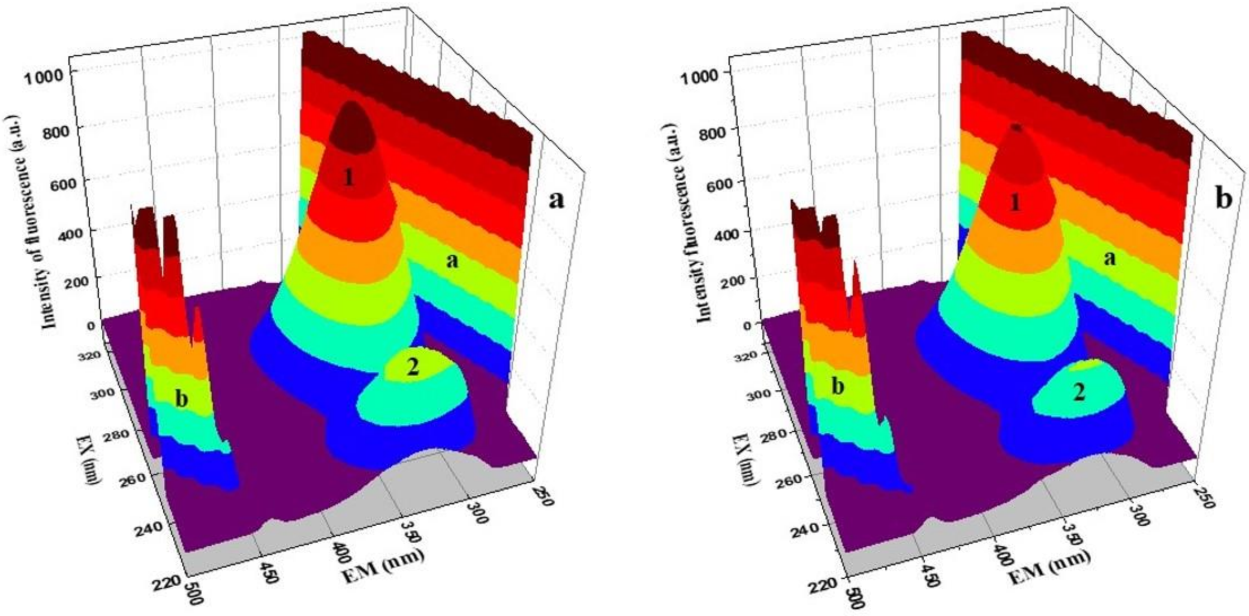

Figure S14 3D fluorescence spectra of HSA (**a**) and EPX/HSA 16/1 (**b**) complex

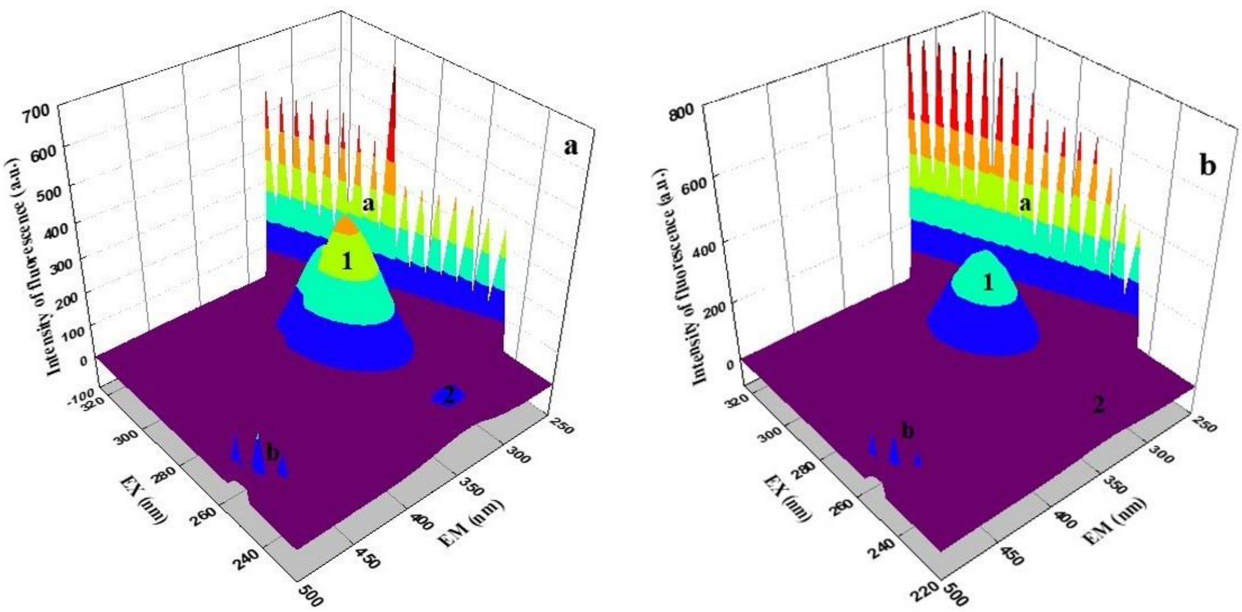

Figure S15 3D fluorescence spectra of BSA (**a**) and EPX/BSA 16/1 (**b**) complex

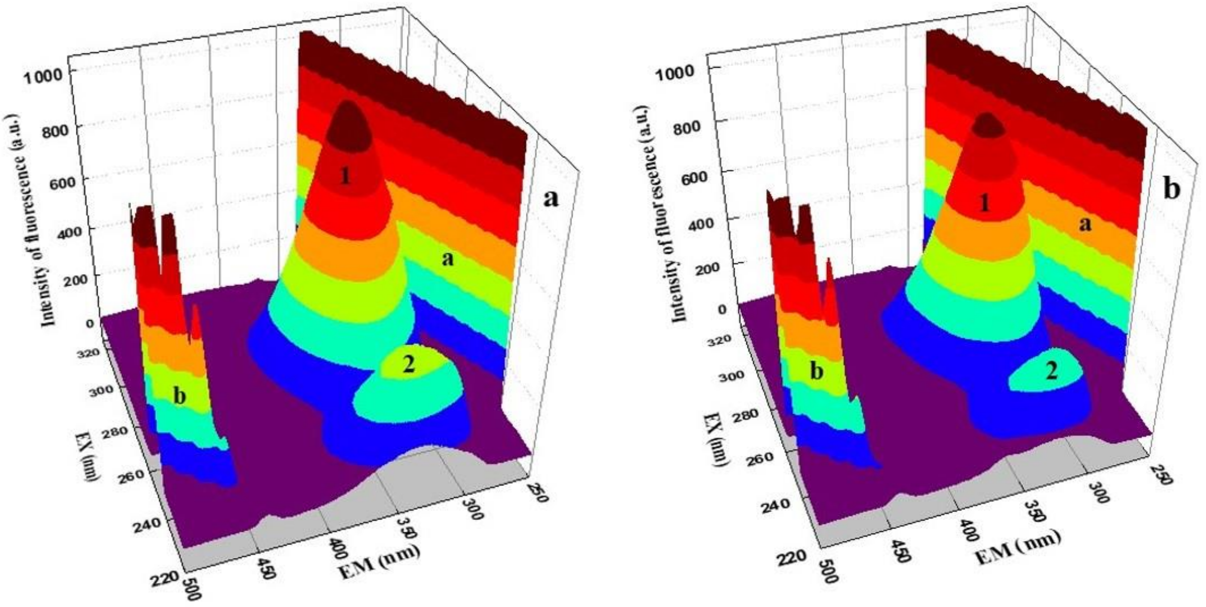

Figure S16 3D fluorescence spectra of HSA (**a**) and PTC/HSA 16/1 (**b**) complex

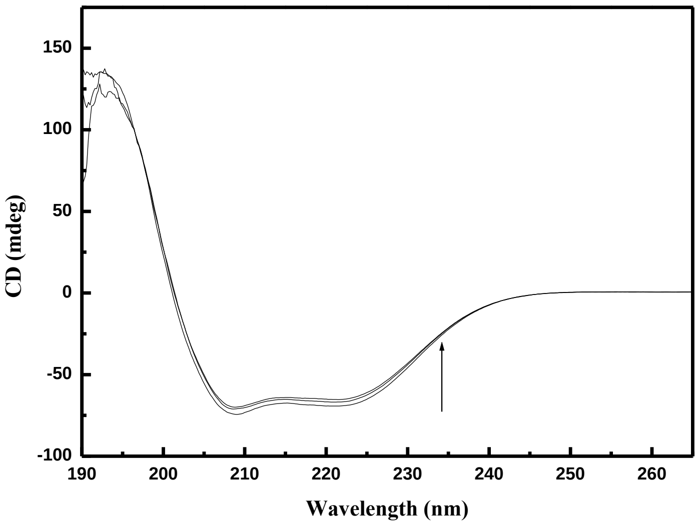

Figure S17 CD spectra of HSA in the presence of EPX

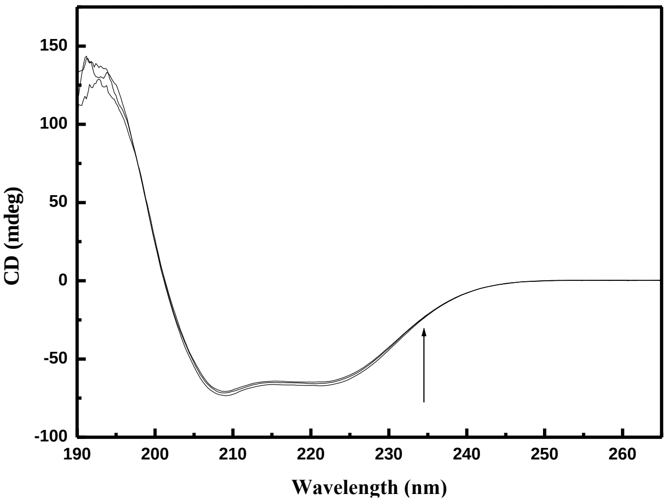

Figure S18 CD spectra of BSA in the presence of EPX

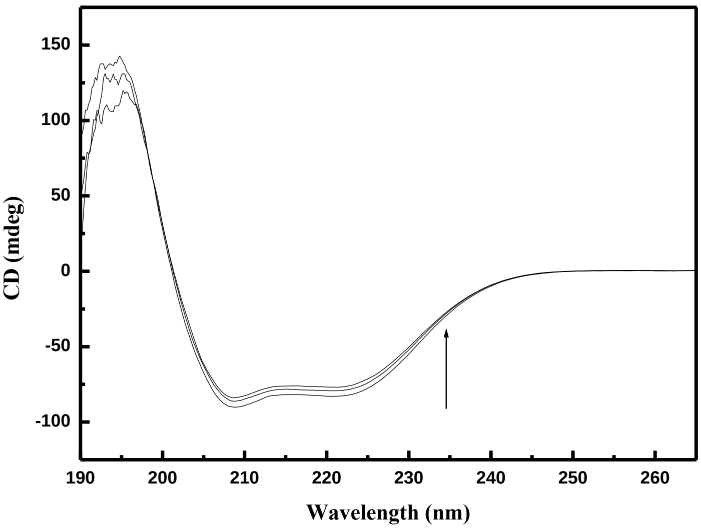

Figure S19 CD spectra of HSA in the presence of PTC
